# Observations on the Difficulty of Ascertaining the Virtues of Medicines

**Published:** 1803-01-01

**Authors:** 


### *Observations on the Difficulty of ascertaining the Virtues of Medicines.*

TO appreciate the value of remedies, and to determine the efficacy of those which popular experience has introduced, or which art has invented, would appear to be a matter of little difficulty; daily experience, however, proves that this opinion is ill founded. Let us consider for a moment, the fluctuating state of what is called the Materia Medica; we shall find that medicines which at one time were dignified with the title of Specific, have sunk into oblivion even before their panegyrists; new medicines have been introduced; and old ones have been revived with equally great promises of infallibility. It affords rather a discouraging prospect to the advancement of our art, when we reflect, that in all ages, Physicians have boasted of their extraordinary cures; and that these were performed by remedies which their successors have treated with indifference or neglect. The sanguine hopes of youth are sported with in no art so much as in that of Medicine; for every practitioner will recollect, how often, in his early practice, he flattered himself with the idea of having discovered a specific, and found that he only grasped a shadow. The contradictory reports respecting the effects of nitrous acid in syphilis, do not impeach the veracity of any person; they merely shew the difficulty and uncertainty of our art, and point out the necessity of caution. No remedy could be recommended by stronger or more respectable testimony than digitalis has been for the cure of phthisis; yet every candid practitioner must allow that he has been too frequently disappointed in its use. In this insidious and fatal disease, innumerable medicines have been celebrated for their virtues; yet, at this moment, we know no more of its cure than was known in the days of HIPPOCRATES.

In the last Number of the Physical Journal is contained an Analysis of a Treatise on Consumption, by Dr. REGNAULT, in which the use of the lichen islandicus, eatable or eryngo leaved lichen, is recommended with much confidence. This remedy, though it has obtained a place in the Edinburgh Dispensatory, has not been noticed by any late writers in this country, except by Dr. CRICHTON and Dr. WOODVILLE; and it has been very rarely employed. Upon the Continent it is a favorite remedy; and is daily employed in the routine of phthisical cases. I shall not here here mention testimonials of its efficacy, extracted from the numerous treatises published upon it in Germany, lest I repeat Dr. REGNAULT's quotations; but shall merely confine myself to a few practical remarks. According to BERGIUS, in its recent state, the lichen islandicus is 'eccoprotica,' and in a dried state, 'nutriens, pectoralis.' In the Dispensatorium Fuldense, it is said to be 'astringens, roborans, humectans, inviscans nutriens, antiseptica.' This moss was first recommended by LINNE, Amœn. Acad. as a popular remedy used in Sweden for coughs. It is not used upon the Continent, indiscriminately, in every species of phthisis, nor in every stage of that disorder. It is chiefly recommended in those instances where the cough is attended with purulent expectoration; in cases preceded by, or accompanied with hæmoptysis; in incipient phthisis, where from relaxation there is an increased discharge of mucus from the bronchia; in the sequelæ of measles, attended with a quick, small pulse, pain of the breast, emaciation, violent cough, and purulent expectoration. The use of this lichen is forbidden when vomicæ are already formed and proceeding towards ulceration; in dyspnœa, and where there is an increased action of the vessels with diminished expectoration. Neither has the lichen islandicus been confined to phthisical cases; it has been recommended in malignant fevers, dysentery, and hæmatemesis; as an enema in hæmorrhoids; as an injection in gonorrhœa, &c. &c.

The purgative quality of this lichen does not appear to be considerable, and depends upon a volatile principle, which is dissipated by drying; indeed, its purgative nature may justly be called in question, as the natives of Iceland, who use it for food, do not complain of this effect; and a case is quoted by BERGIUS, in which it restrained, for a considerable time, an obstinate diarrhœa. In the recent state, however, it is said to be boiled in milk and frequently used by the peasants to purge and expel worms. The most usual form of exhibiting this remedy, is a decoction of it in milk, or when this disagrees with the stomach, in water. I have scarcely met with any instances, in authors, where the cure of phthisis has been solely trusted to this remedy; but in general it has been united with other active medicines, as squills, opium, cinchona, &c.

Dr. I. G. FRITZE of Berlin, a celebrated and experienced physician, notices the Iceland moss, in his Medizinisch. Annalen, and recommends it as a valuable remedy in phthisis; but he limits its use to those cases in which pus is expectorexpectorated. To this excellent medicine, he adds, several phthisical patients under his care, have been, for some years, indebted for a tolerable degree of health. He generally exhibited a decoction of it in milk, or when this was disagreeable to the stomach, in water, to which were added the flowers of the hyperic. perforat. et tussilago farfara.

In the Annalen des Klinischen Instituts zu Berlin, by Dr. I. F. Fritze, Professor of Medicine, several cases of phthisis are related, in which this moss was tried. The success does not appear very flattering, nor was the remedy trusted to alone, but several potent auxiliaries were called in, as blisters, issues, antimonial preparations, sal ammoniac, dulcamara, g. ammon. polygala, senega, &c. Where the indication, he observes, is to strengthen the organs of digestion without irritation, to nourish the body, and to supply it with bland fluids, the Iceland moss will be found extremely useful. In his practice he has ordered from three to four ounces, or more, of this jelly to be consumed daily; but when the patient's stomach cannot bear so much, he advises us to lay it aside, as it will be of no use in a smaller quantity. There is so much candour in the following Extract from Dr. Schaeffer's Ortbeschreibung der Stadt Regensburg, that I cannot forbear inserting it. "As this disease (phthisis) occurred here this year, 1786, more frequently than usual, I had an opportunity of trying the usual remedies; but alas! I did not cure one of my patients. I was successful only in those cases, where the patients called in time for assistance; where the fever was moderate, and the disease had not yet fixed itself upon the lungs." His usual practice was to give every second or third day such a dose of ipecac. as excited vomiting two or three times; and in the intermediate days to exhibit the Iceland moss, valerian, polygala, amara, g. arabic, the bark, &c. But when vomicæ were formed and in an inflamed or suppurating state, with considerable fever, there the bark was evidently hurtful. This mode of cure, he adds, was only useful at the commencement of the disease; for when this first period was neglected, as in most instances where the disease had been considered merely as a common cold, not only was the above plan of no avail, but also the following remedies, viz. the expressed juice of plants, wild strawberries, the *juice of cucumbers*, butter-milk, Seltzer water mixed with goat's milk, the watery extract of myrrh, &c. &c.

It does not appear to me that the Iceland moss will ever become become a favourite remedy with English Practitioners, though it certainly merits a further trial. It owes much of its celebrity to the prevalence of the humoral pathology; for, as it consists of a mucilage united with a bitter, it was supposed that the first principle would render it powerful in inviscating acrimony; and the second in gently constringing and corroborating the muscular fibres of the stomach. The bitter principle however, which has been considered of such importance, cannot be very strong or durable, as it is easily extracted by a slight maceration in cold water, the only process requisite before it is prepared for food. The mucilage is contained in considerable quantity, and it is said to thicken milk or water as much as three times the same weight of gum arabic, but perhaps not more than an equal quantity of linseed. This latter substance, which, as a demulcent, is equal to any we possess, is said to afford but little nourishment, to produce flatulency, and weaken the digestive powers. But in the instance quoted by Dr. Woodville, Medical Botany, Vol. II. p. 305, it is not improbable that boiled linseed constituted the *whole diet* of the people of Zealand during a scarcity of long continuance; in which case it is not to be wondered at that symptoms of debility should occur, attended with "a remarkable distension of the hypochondria, swellings of the face and other parts, which in several instances proved fatal." Perhaps the same effects would arise from the Iceland moss, were it to constitute the sole article of diet for any length of time. We know that caravans have been supported for a considerable time upon gum arabic alone, or occasionally mixed with a little camel's milk, but we cannot suppose that such a diet could be supported without inconvenience. We possess, however, several vegetable substances which yield a mucilage more agreeable to the taste than linseed, and which afford as much nutriment, and of as easy digestion, as the Iceland moss. The chief of these are sago, tapioca, Indian arrowroot, and particularly salep; half an ounce of the latter substance, boiled in a quart of milk, yields an agreeable and nourishing mixture, not inferior to any we can procure. Dr. Fritze, in his Medical Annals, speaks highly of the salep, which his experience, he adds, has shewn him to be nearly as efficacious as the Lichen Islandicus. He frequently prescribed it in cases of phthisis, attended with a dry cough, or in which the expectoration was restrained by some acrid matter; the following is the formula which he generally used: ℞. Rad.

℞. Rad. saleb. ℥iss. Granor. sago, ℥j. gum tragacanth. ʒj. M. ft. pulv.

A few tea spoons full of this powder was mixed with warm water and milk, to the consistence of a thick pap, and used night and morning. When the salep was boiled, the lichen islandicus was added to it; and in both modes of preparation, he assures us, it shewed more soothing and healing powers than any balsamics, or than the best preparation of opium. Professor FRITZE observes, that the decoction of salep, and other vegetable mucilages, were peculiarly useful in the more violent stages of hectic fever; in an inflammatory state of the system, or in that of a single viscus. But where the irritation which kept up the fever, partook of a bilious, rheumatic, herpetic, or scrophulous acrimony, they were inferior to the lichen and *boletus suaveolens*, because the two latter substances at the same time strengthen the organs of digestion; whereas the former, when used daily in large quantities, rather relaxed those organs, and at the same time had a tendency to produce a viscid mucus and a morbid acid in the alimentary canal. With regard to the substance last mentioned, boletus suaveolens *(Weidenschwamm)*, it is used in phthisis with the same view as the lichen. Prof. FRITZE observes, that it produced excellent effects in several kinds of hectic, and in the phthisis pituitosa; but in an ulcerated state of the lungs, connected with an inflammatory disposition, or congestion in the breast, this substance, as well as the lichen, exasperated the symptoms. The good effects of both these remedies, he continues, in diseases of the lungs, depend upon their gently astringent, mucilaginous, and restorative qualities. In its sensible qualities, the boletus stands between the cinchona and lichen; it is more mucilaginous than the former, and possesses more of an astringent and volatile principle than the latter. It is given in doses of, from ʒij to ℥j daily, in powder or electuary; when used in decoction, ℥ij of the boletus must be boiled in a sufficient quantity of water, down to ℥vj.

Though it appears probable that the lichen islandicus will never be much employed in this country as a medicine, yet in an œconomical point of view, it merits peculiar attention. In times of scarcity, such as we have lately experienced, this moss, either alone or mixed with a certain proportion of farinaceous matter, offers an useful substitute for bread. In the country from which it receives its trivial name, this lichen constitutes a considerable portion of the diet of the inhabitants; and the harvest of moss is attended to with as much care as that of wheat in this country. The learned VAN TROIL, in his Letters on Iceland, observes, that a flour is obtained from the *fiálgras* (rock grass) L. Island. by washing it, and then cutting it into small pieces, "though the greater number, (he adds) dry it by the fire or sun, then put it into a bag, in which it is well beaten, and, lastly, worked into flour by stamping." It seems probable that this moss has never been used as an article of diet in this country, though it grows abundantly in those parts where the cerealia do not thrive, as in the mountainous parts of Wales, and on the barren rocks, &c. in the Highlands of Scotland. It was formerly supposed to possess eminent virtues in diseases of the liver, hence it obtained the popular name, Liverwort. Dr. REES, in his edition of Chambers's Dictionary, observes, that in the county of Glamorgan, a kind of bread, called laver bread, is made from the sea liverwort; but this belongs to a different family of plants (*Ulva*).

The juice of raw cucumbers has been mentioned above in the list of remedies used in phthisis; and as it may excite a little curiosity, I shall offer a few remarks upon it. This remedy was first employed by the celebrated MUZELL, of Berlin, in the case of a young man who had several times laboured under hæmoptysis; this was at length checked by three large bleedings, but was succeeded by hectic and great emaciation. The pulse was constantly quick; hot and chilly fits came on after meals, which terminated during the night in sweat. The cough was severe, and purulent matter was expectorated. His strength and flesh continued to waste; the dyspnœa was so great that he was obliged to be raised very high in bed, and immediately lost his breath on attempting to stand erect, or to use the least motion. Being tired of medicines, and milk disagreeing with him, the use of fresh cucumbers was proposed by his physician. Of these he was not limited to any number, but ordered to eat as many, without the skins, as he chose. He was allowed only biscuit, and water for his drink; and by pursuing this plan he obtained a complete cure. Dr. J. G. FRITZE speaks of this remedy in terms of high commendation, and observes, that many phthisical patients are indebted to it for their recovery. He adds, that by this means he has *here* and *there* snatched a patient from approaching death, where every other method had been tried in vain. In those cases where it did not succeed, it still procured considerable relief, by abating the violence of febrile action, quenching the thirst, and improving the quality of the matter expectorated.

*Nov.* 20, 1802. TYNICOLA.

